# *Streptococcus pneumoniae* Serotype Distribution and Pneumococcal Conjugate Vaccine Serotype Coverage among Pediatric Patients in East and Southeast Asia, 2000–2014: a Pooled Data Analysis

**DOI:** 10.3390/vaccines4010004

**Published:** 2016-02-22

**Authors:** Stanley S. Tai

**Affiliations:** Department of Microbiology, College of Medicine, Howard University, Washington, DC 20059, USA; stai@howard.edu; Tel.: +1-202-806-4677; Fax: +1-202-806-4508

**Keywords:** *Streptococcus pneumoniae*, vaccine, Asia

## Abstract

Pneumococcal infection is one of the leading causes of death worldwide, especially in children of developing and underdeveloped countries. Capsular polysaccharide-based vaccines are available for the prevention of this disease. A 7-valent pneumococcal conjugate vaccine (PCV7) was licensed in 2000 for use in children less than two years of age. Subsequently, to broaden the protection, 10-valent (PCV10) and 13-valent (PCV13) vaccines were licensed in 2009 and 2010, respectively. All of these conjugate vaccines elicit an immune response that only provides protection against the infection of *S. pneumoniae* serotypes included in the formulation. Profiles of *S. pneumoniae* serotype distribution and serotype coverage for both PCV7 and PCV13 have been reported in some Asian countries/territories. But the published results cannot provide conclusive information due to the difference in studied population and geographic areas. The goals of this review are to obtain an accurate estimate of serotype coverage for PCV7, PCV10, and PCV13 and examine the change in the *S. pneumoniae* serotype distribution after PCV7 use among pediatric patients in East and Southeast Asia through the analysis of pooled data that were published in the English literature between 2000 and 2014.

## 1. Introduction

*Streptococcus pneumoniae* infection leads to many clinical manifestations including meningitis, septicemia, bacteremia, pneumonia, acute otitis media, and sinusitis. It has caused great mobility and mortality worldwide, especially in children. Pneumococcal infection annually has caused approximately 14.5 million cases of invasive pneumococcal disease (IPD) and 0.7–1 million deaths in children under five years old, mostly in developing and underdeveloped countries [1]. Recent data estimate that the incidence rate of pneumococcal disease among children in the Asia-Pacific region from 1999–2010 is 100–200 cases per 100,000 children younger than two years old [2]. The concerns of high mortality rate, rapid increase of antibiotic resistance in *S. pneumoniae*, and heavy burden on the health care and welfare systems have called for efficacious vaccines against pneumococcal infection.

At least 93 different serotypes of *S. pneumoniae* have been identified based on the cross-reactivity of antibodies against capsular polysaccharides. Though only a limited set of serotypes are frequently isolated from pneumococcal patients, the development of a universal capsular polysaccharide-based vaccine against all *S. pneumoniae* infection is hindered by the specificity of antibodies, the variations of prevalent serotypes among different geographic areas, and demographic populations. A 23-valent pneumococcal polysaccharide vaccine (PPSV23), which contained the capsular polysaccharide of 23 most predominant serotypes that accounted for 88% of IPD cases, was developed in 1983 [3]. It elicits a T-cell independent immune response and has the efficacy of 56%–81% in various clinical trials [4]. It is most effective in adults, but is not effective in children younger than two years old. The success of *Haemophilus influenzae* type b vaccine led to the development of a heptavalent pneumococcal conjugate vaccine (PCV7) to provide protection against the infection of seven serotypes (4, 6B, 9V, 14, 18C, 19F, and 23 F) that were responsible for approximate 80% of IPD cases in young children in the United States [5]. This vaccine was licensed in 2000 by the U.S. Food and Drug Administration and recommended for use in children under two years old [5]. Unlike PPSV23, PCV7 elicits a T-cell dependent immune response and has great success in controlling *S. pneumoniae* infection. PCV7 use results in a significant reduction in the incidence rates of IPD and acute otitis media in vaccinated children [6,7,8,9]. Via herd immunity, PCV7 immunization also reduces the pneumococcal colonization and IPD in the non-vaccinated populations [10,11]. However, the effectiveness of PCV7 has created a new challenge: an increase of non-vaccine serotypes *S. pneumoniae* in pediatric patients [11,12,13,14]. For broader protection, vaccines including more serotypes were licensed: 10-valent PCV (PCV10) containing 3 additional serotypes (1, 5, and 7F) in 2009 and 13-valent PCV (PCV13) containing 6 additional serotypes (1, 3, 5, 6A, 7F, and 19A) in 2010 [15,16], respectively.

PCV7, PCV10, and PCV13 have been gradually introduced into Asia countries/territories. Their efficacies in this region have not been reported but have been estimated based on the predicted vaccine serotype coverage. Profiles of *S. pneumoniae* serotype distribution and serotype coverage for both PCV7 and PCV13 in some Asian countries/territories have been published. But most of the published studies were conducted by individual investigators or by collaboration of hospitals. Data of national surveillance are only available in a limited number of countries. It is difficult to get conclusive information due to considerable variations among these reports in their sample sizes, study periods, geographic area, and health status and age of studied population. For example, serotypes that are commonly found in adults may not be as prevalent in children. The serotype profiles of *S. pneumoniae* isolated from the nasopharynx of healthy children are different from those collected from the blood, cerebrospinal fluid, or other sterile body fluid of children suffering from pneumococcal disease. Studies on different populations may lead to different conclusions. Results generated from the studies of small numbers of pneumococcal isolates or only focused on specific geographic regions would be misleading if they were extrapolated as a representative of the entire country. To get a better understanding of the effect of PCV7, differing from previous reviews that described the results of individual studies [2,17,18,19,20,21], this communication focused on the serotype distribution and serotype coverage for PCV7, PCV10, and PCV13 in children suffering from pneumococcal disease before and after the use of PCV7 in countries/territories of East and Southeast Asia. The serological typing results of all *S. pneumoniae* specimens isolated from pediatric patients (<18 years old) and published in the English literature between 2000 and 2014 were collected, combined, and analyzed. The pooled data provides information representing isolates from a broader geographic area and a larger sample size of PCV7-intended population (children). The change in the serotype distribution and vaccine serotype coverage after PCV7 use in countries of this region was examined.

## 2. Data Collection

Publications on *S. pneumoniae* serotypes distribution were identified by search of MedLines database (http://www.ncbi.nlm.nih.gov/pubmed/). In combination with country names, search terms included “*Streptococcus pneumoniae*”, “pneumococcus”, “serotypes”, and “pneumococcal”. Nineteen countries/territories in East and Southeast Asia were included in the search, in alphabetical order: Brunei, Cambodia, China, East Timor, Hong Kong, Indonesia, Japan, North Korea, South Korea, Laos, Malaysia, Macau, Mongolia, Myanmar, Philippines, Singapore, Taiwan, Thailand, and Vietnam. Primary papers published in English between 2000 and 2014 were collected and further screened.

Since PCV7 was recommended for use in children, we only focused on both invasive and noninvasive *S. pneumoniae* that were collected from patients aged ≤18 years. The invasive isolates were obtained from blood, pleural fluid, cerebrospinal fluid, and joint fluid; the noninvasive isolates were from pharynx, middle ear fluid, sputum, eye, and purulent discharge. Specimens that were collected from adults, unspecified age groups or healthy children, or for studies that were focused on a particular characteristic of *S. pneumoniae*, such as antibiotic resistance, were excluded. The serotype information in all articles was compiled and organized according to the date of isolation before or after the use of PCV7 for each of the countries/territories. Data for the isolates whose date of isolation could not be determined were excluded. The Microsoft Office Excel program was used to organize and recalculate these data. Most studies reported their typing results in serotypes but some only differentiated their isolates at the serogroup level. To overcome this difference in calculation, the serotype distributions of the un-serotyped serogroups were estimated based on the assumption that their serotypes were distributed at the same ratio as those in other studies of the same country in which the serotypes had been determined. The serotype distribution was calculated by dividing the number of isolates for each serotype with the total number of isolates in each country and adjusted to percentage. The predominant serotype was defined as the serotype that had a distribution percentage greater than 5% of the total isolates. The vaccine serotype coverage was the sum of the percentages of all serotypes in the respective vaccine, PCV7, PCV10, or PCV13. In some studies, minor serotypes/groups were categorized as “others”. Although the definition of “others” might be different among these studies, the distributions of “others” were small and had a negligible effect in the calculation of vaccine coverage.

## 3. Serotype Distribution and Pneumococcal Conjugate Vaccine Coverage

To reduce the bias in information caused by the difference in geographic areas and specimen numbers, this review focused on the countries/territories that had serotype information for more than 200 *S. pneumoniae* isolates and was published in at least two independent reports. Among 19 countries/territories in East and Southeast Asia, there were no reports from Brunei, Cambodia, East Timor, Macau, Mongolia, Myanmar, and North Korea; one from Indonesia (11 isolates), Laos (42 isolates), and the Philippines (57 isolates); and two from Malaysia (157 isolates) and Vietnam (137 isolates). Only seven countries/territories had met the selection criteria and were included in the analysis. They were China, Hong Kong, Japan, Singapore, South Korea, Taiwan, and Thailand. Table 1 lists the relevant information of individual studies on *S. pneumoniae* collected from pediatric patients in these seven countries/territories. The profiles of predominant serotypes and serotype coverage for PCV7, PCV10, and PCV13 in both the pre-and post-PCV7 periods are presented in Table 2 and Table 3, respectively. The detailed serotype distribution of *S. pneumoniae* in these seven countries is shown in the supplementary data.

### 3.1. China

Eleven studies on *S. pneumoniae* serotype distribution in pediatric patients were published between 2000 and 2014 [22,23,24,25,26,27,28,29,30,31,32]. Seven were conducted on the specimens collected before PCV7 was introduced into the market in 2008. Results of individual studies in the pre-PCV7 period showed a high degree of variations in vaccine serotype coverage, ranging from 57%–81% for PCV7 and from 79.6%–92.3% for PCV13, respectively. Pooled results showed that serotype coverage for PCV7 and PCV13 were 57.6% and 70.7%, respectively. There were five predominant serotypes, 19F, 23F, 6B, 19A, and 14, and four of them were covered in the PCV7 formulation. Five years after the introduction of PCV7, vaccine serotype coverage did not show a reduction but an increase. It was increased to 60.5% for PCV7 and 79.8% for PCV13, respectively. The change was mainly due to the increase of distribution in serotypes, 6B, 9V, 14, and 23F. The predominant serotypes remained the same but the order of prevalence changed in the post-PCV7 period. Serotype 19F was the most frequently isolated serotype in both the pre-and post-PCV7 periods. Serotype 19A was already prevalent in the pre-PCV7 period and its increase in distribution in the post-PCV7 period was the major contributor for the increase in PCV13 coverage. The distribution of serogroup 15, a non-PCV13 specific serotype, had increased to 5.6% and became prevalent in the post-PCV7 period. It was noted that PCV7 serotype coverage in the pre-PCV7 period was higher in the cities of eastern and southern China than in those in the north [28]. A similar phenomenon was also observed in the post-PCV7 period when comparing Beijing, Shenyang and Shenzhen, Suzhon [29,30,31,32]. The difference in serotype distribution between northern and southern cities may contribute to the variations of PCV7 serotype coverage in the published reports and affect the effectiveness of the vaccine, if it is used.

### 3.2. Hong Kong

PCV7 has been available in the market since October 2005 and included in the childhood immunization program since September 2009 [35]. Five articles on *S. pneumoniae* serotype distribution were published between 2000 and 2014 [33,34,35,36,37]. Based on the dates of collection, four sets of data each could be extracted from these publications for the isolates in pre- and post-PCV7 periods, respectively. Comparison of the pooled data from these two periods has shown significant changes in serotype distribution and vaccine serotype coverage after the use of PCV7. The distribution of all PCV7 serotypes was reduced, except serotype 19F, which increased from 10.1%–16.6%. The most dramatic reduction was in the distribution of serotype 14. It was the most predominant serotype in the pre-PCV7 period, accounting for 34% of the total isolates, and its distribution decreased to 10% in the post-PCV7 period. This decline was a major contributor to the reduction in vaccine serotype coverage, from 89.2%–55.6% for PCV7, 89.2%–55.6% for PCV10, and from 91.3%–66.1% for PCV13, respectively. Other changes in the post-PCV7 period included the reduction in number of predominant serotypes from five to four and the increase in the distribution of both PCV13 specific serotypes, 6A and 19A, and non-PCV13 serotypes, especially the non-typable *S. pneumoniae*. No new predominant serotype has emerged in the post-PCV7 period. Unlike other Asian countries, 19A was not a predominant serotype in Hong Kong.

### 3.3. Japan

Japan has well-established national surveillance networks that periodically monitor the serotype distribution and antibiotic resistance of *S. pneumoniae* collected from children with meningitis, acute otitis media, IPD, or respiratory tract disease. PCV7 was approved for voluntary use in March 2010 [44,47]. The majority of published reports, including six nationwide surveillance and six individual studies, were conducted on the *S. pneumoniae* collected in the pre-PCV7 period [38,39,40,41,42,43,44,45,46,47,48,49]. The PCV7 and PCV13 serotype coverage in these reports varied greatly, ranging between 20.0% and 81.3% for PCV7 and 30% and 100% for PCV13. Pooled results showed that five serotypes were predominant (>5%) and four of them were components of PCV7. PCV7 and PCV13 covered 68.6% and 83.5% of total isolates, respectively. Two studies were conducted on the isolates collected in the post-PCV7 period. Though the sample sizes were small, results of both studies showed a yearly decrease in PCV7 serotype coverage as the vaccination rate increased and the coverage dropped to 11.8% and 14.7%, respectively, at the end of each study [50,51]. Combining all data in the post-PCV7 period showed that serotype coverage for PCV7 and PCV13 was 53.1% and 71.9%, respectively, and the isolation frequency of all predominant serotypes had reduced except 6B. Serotype 6B was the most frequent isolate in both pre- and post-PCV7 periods. The use of PCV7 also led to the increase of distribution of serotype 19A and non-PCV13 serotypes, such as 6C and 15A. Serotype 19A emerged as a new predominant serotype after the introduction of PCV7.

### 3.4. Singapore

PCV7 has been available for optional use since October 2005 and was incorporated into the national children’s vaccination program in November 2009 [75]. Two studies that focused on the *S. pneumoniae* serotype distribution among pediatric patients in the pre-PCV7 period were reported between 2000 and 2014 [52,53]. Pooled data showed that the predominant serotypes accounted for 84.1% of total isolates, with serotype 19F being the most frequent isolate at 31.1%. PCV7, PCV10, and PCV13 covered 85.7%, 86.1%, and 93.0% of isolates, respectively. No information on the *S. pneumoniae* serotype distribution in pediatric patients in the post-PCV7 period was available.

### 3.5. South Korea

PCV7 was licensed for optional use in South Korea in 2003, the first among all Asian countries. Six articles on the serotype distribution of *S. pneumoniae* among pediatric patients have been published and three sets of data could be extracted from these publications for the isolates collected in the pre-PCV7 period and nine in the post-PCV7 period [55,56,57,58,59,60]. The PCV7 serotype coverage for the pre-PCV7 period in these reports ranged between 56.4% and 80.3%, and for the post-PCV7 period, between 16.5% and 62.3%, respectively. Pooled data of isolates in the pre-PCV7 period showed that seven serotypes were predominant (>5%) and five of them were components of PCV7. PCV7 serotype coverage was 64.5% and the majority of it came from the distribution of serotypes 23F and 19; each accounted for 21% of the total isolates. Seven years after the introduction of PCV7, serotype distribution became more diversified and serotype coverage for PCV7 dropped to 34.6%. Serotypes 9V and 14 were no longer predominant and 19A became the most prevalent serotype in the post-PCV7 period. The distribution of non-PCV13 serotypes increased to 38.3%, especially the non-typable *S. pneumoniae*, which accounted for 11.9% of the total isolates. The serotype coverage for PCV10 and PCV13 in pediatric patients in the post-PCV7 period was 35.5% and 61.7%, respectively. Both PCV10 and PCV13 were introduced for optional use in 2010. Their effects on the *S. pneumoniae* serotype distribution have not reported. It should be noted that the majority of *S. pneumoniae* strains in these studies were collected in Seoul and adjacent areas. The conclusion might not be applicable to the child population of the entire country.

### 3.6. Taiwan

PCV7 has been available in the market for optional use since 2005. A total of seven articles covering the serotype information of *S. pneumonia* from pediatric patients were published between 2000 and 2014 [61,62,63,64,65,66,67]. Two of them were for the isolates collected in the pre-PCV7 and three in the post-PCV7 period. Information for specimens collected in the transition period was not included in the analysis [64]. Pooled data of isolates in the pre-PCV7 period showed that all four predominant serotypes, 23F, 14, 19F, and 6B, were components of PCV7 and the serotype coverage for PCV7 was 87.1%. Seven years after the introduction of PCV7, the coverage was reduced to 54.1%, however the reduction in PCV7 serotype coverage had little effect on the serotype coverage of PCV13, which showed a slight change from 92%–89.5%. The decrease of PCV7 serotype distribution was compensated for by the increase of PCV13 specific serotypes, 19A and 3. Serotype 19A emerged as the most predominant serotype in the post-PCV7 period. The number of predominant serotypes increased from four to six after the use of PCV7.

### 3.7. Thailand

PCV7 has been available for optional use in Thailand since 2006. Seven articles on the serotype distribution of *S. pneumoniae* in pediatric patients have been published and four sets of data could be extracted from these publications for the isolates in the pre-PCV7 period and three sets for the post-PCV7 period [68,69,70,71,72,73,74]. Information for the isolates collected in the transition period was not included in the analysis [71,72]. Pooled data showed that serotypes 6B, 23F, 19F, and 14 were predominant in both periods and the distribution of serotype 19A had increased and emerged as a new predominant serotype in the post-PCV7 period. The serotype coverage for PCV7, PCV10, and PCV13 in the pre-PCV7 period was 74.1%, 76.6%, and 81.8%, respectively. It showed little changes in the post-PCV7 period. This might be due to the low vaccination rate in the country [73]. It should be noted that the majority of studies were focused on PCV7 serotypes. The details of serotypes of non-PCV7 vaccine types, which have contributed 18% and 15% of the total isolates, respectively, in the pre- and post-PCV7 period, were not determined in the literature. Thus, the vaccine coverage for PCV10 and PCV13 might be underestimated.

## 4. Conclusions

Since PCV7 was licensed in the U.S. in 2000 it has been slowly introduced into countries/territories in East and Southeast Asia. Its impact on *S. pneumoniae* serotype distribution in these countries has been reported. But the difference in study methods has resulted in various conclusions. To avoid the bias in sample sizes, age, and health status of the studied population and to accurately evaluate the effect of PCV7 use, this review focused on the serotype distribution of seven countries/territories in East and Southeast Asia that had at least two independent reports and had information for more than 200 isolates collected from pediatric patients. Pooled data showed considerable differences in the *S. pneumoniae* serotype distribution and vaccine serotype coverage among these selected countries/territories. Not all but four of the PCV7 serotypes, 6B, 14, 19F, and 23F were predominant in all seven countries in the pre-PCV7 period. Serotype 9V was only predominant in South Korea and serotype 18C only in Hong Kong. Serotype 4 was not frequently isolated in all seven countries. The difference in the distribution of predominant serotypes among these countries was reflected in their vaccine serotype coverage. Before the introduction of PCV7, the serotype coverage for PCV7 in Hong Kong, Singapore, and Taiwan was greater than 80%; Japan, South Korea, and Thailand between 80% and 60%, and China below 60%. It is perceivable that efficacy of PCV7 in these countries would be varied and some would be lower than that in the United States and other countries that had 80% of vaccine serotype coverage in the pre-PCV7 period.

The data also showed a difference in the pattern of changes in vaccine serotype coverage and profile of predominant serotypes among these countries after the introduction of PCV7. The PCV7 serotype coverage was reduced 30% in South Korea and 32% in Taiwan after seven years of vaccine use, 34% in Hong Kong after five years, 6% in Thailand after four years, 16% in Japan after three years, and remained unchanged in China. Changes in the distribution of each PCV7 serotype after PCV7 use were varied among these countries/territories. The distribution of serotypes 14 and 23F isolates showed the most reduction in Taiwan (16.6% and 17.9%,), followed by South Korea (3.7% and 16%), Hong Kong (2.4% and 7%), and Japan (2.9% and 3.5%), respectively, but increased in China and Thailand. Serotype 19F had a moderate reduction in South Korea (8.6%), Japan (7.4%), China (4.4%), and Taiwan (3.4%), but increased in Hong Kong and Thailand. Serotype 6B had the least reduction among all PCV7 components. The change of predominant serotype profile after the introduction of PCV7 was also noticeable. Serotypes 6B, 19F, and 23F remained prevalent in all seven countries/territories. Serotype 18C was no longer prevalent in Hong Kong, nor serotype 6A in Japan, and serotypes 9V and 14 in South Korea. The use of PCV7 also led to an increase in the distribution of non-PCV7 serotypes which resulted in the emergence of new predominant serotypes in some countries, such as serotype 19A in Japan, Taiwan, and Thailand, serotype 3 in Taiwan, and serogroup 15 in China and Japan. Non-typable *S. pneumoniae* have become prevalent in the post-PCV7 period in Hong Kong and South Korea. It is not clear what factors contribute to the various changes in these countries after the use of PCV7. They may include the difference in vaccination rate, the use of antibiotics, the presence of antibiotic-resistant strains, immunogenicity of each conjugate in various populations, and the possibility of the presence of serotype variants in some countries that were different from the strain used in the vaccine preparation. Further analysis is required to elucidate how these factors interplay to determine the effect of vaccine use.

PCV is effective in protecting the infection of vaccine serotype and the vaccine serotype coverage could be used as a predictor for vaccine efficacy. PCV13 has been licensed to use as a replacement for PCV7. Its predicted efficacy in pediatric patients of these seven East and Southeast Asian countries/territories ranged between 90% and 62%. Based on the analysis of this report, the predicted PCV13 efficacy would further decrease as the PCV7 vaccination rate increased and serotype distribution showed greater variations. It may not be cost effective to use PCV13 for countries that have low predicted efficacy and low distribution of PCV13 specific serotypes. The major drawback of PCV is the need for periodical revision of vaccine formulation to include more serotypes to provide broader protection. However, selection of a serotype in the new version of PCV is a complicated process. Besides the need for updated information on serotype distribution, other factors are also considered, such as the immunogenicity of the new component, its interaction with other components of the vaccine, manufacturing difficulties, and results of clinical trials. The cost would be prohibitively high to manufacture a vaccine that includes the predominant serotypes of all regions, especially for developing and underdeveloped countries that have high morbidity and mortality in pediatric pneumococcal disease and lack of infrastructure to conduct national surveillance on *S. pneumoniae* serotype distribution. To overcome these limitations, the most effective approach would be to develop a capsular polysaccharide independent vaccine. Progress has been made on alternative vaccine candidates, such as pneumococcal proteins, recombinant organisms expressing pneumococcal proteins, and attenuated or killed pneumococcal cells. This new generation of pneumococcal vaccine may provide universal protection against infection for all serotypes of *S. pneumoniae*.

## Figures and Tables

**Table 1:** Studies on serotype distribution of *Streptococcus pneumoniae* isolated from Asian pediatric patients, 2000–2014.

Country.	Isolates (*n*)	Age (Years)	Study Period	Location	Disease	Serotype Coverage (%) PCV7 PCV13		Reference
China	50	<5	1997–1998	Beijing	NS ^1^			[22]
	112	<3	2000–2001	Shanghai	RI ^2^	81		[23]
	625	<5	2000–2002	3 cities	RI	57.6		[24]
	519	<5	2000–2005	Beijing	RI	57		[25]
	451	<5	2005–2006	8 cities	NS	63.6	79.6	[26]
	171	<14	2006–2008	11 cities	IPD ^3^	60.3	87.8	[27]
	338	<5	2006–2008	5 cities	Pneumonia	76.3	92.3	[28]
	140	Pediatric	2010	Beijing	RI	43.6	60.0	[29]
	61	<5	2009–2011	Shenyang	IPD	54.1	85.4	[30]
	87	<14	2009–2012	Shenzhen	IPD	77.0	89.7	[31]
	178	<5	2012–2013	Suzhou	IPD	71.9	89.9	[32]
Hong Kong	88	<6	1999–2000		IPD	89.7		[33]
	88	<5	1995–2001		IPD	89.7		[34]
	86	<5	1995–2001		IPD	89.5	93.0	[35]
	16	5–17	1995–2001		IPD	81.3	87.5	[35]
	519	<15	2005–2006		RI	72.3		[36]
	70	<5	2005–2009		IPD	65.7	91.4	[35]
	15	5–17	2005–2009		IPD	60.0	73.3	[35]
	911	<16	2008–2010		RI	44.2	58.6	[37]
Japan	138	≤17	1999–2002	Nationwide	Meningitis	76.1		[38]
	392	pediatric	2002–2004	Nationwide	Pneumonia	70.9	84.9	[39]
	36	1-5	NA2	Wakayama	AOM ^4^			[40]
	175	<11	2006–2007	Nationwide	AOM	60.6	82.9	[41]
	51	<16		Niigata	RI			[42]
	191	≤17	2006–2007	Nationwide	IPD	75.4	93.7	[43]
	63	<16	2008–2009	Chiba City	Pneumonia	66.7	81.0	[44]
	5	<16	2008–2009	Chiba City	Pneumonia	80.0	100	[44]
	16	<15	2007–2009	Niigata	NS	81.3	93.8	[45]
	60	<15	2007–2009	Niigata	Pneumonia	57.1	73.0	[45]
	52	<1	2007–2009	Nationwide	Meningitis	70.6	82.4	[46]
	51	1–5	2007–2009	Nationwide	Meningitis	72.5	80.4	[46]
	12	5–15	2007–2009	Nationwide	Meningitis	20.0	30	[46]
	76	pediatric	2000–2010	Hokkaido	IPD	71.4	83.1	[47]
	241	pediatric	2008–2009	Nationwide	Pneumonia	68.7	80.9	[48]
	24	pediatric	2008–2012	22 cities	IPD			[49]
	300	<18	2010	Nationwide	IPD	73.3	89.0	[50]
	146	<18	2011	Nationwide	IPD	54.8	72.6	[50]
	156	<18	2012	Nationwide	IPD	14.7	39.1	[50]
	19	<5	2008	Chiba	IPD	57.9		[51]
	27	<5	2009	Chiba	IPD	63.0		[51]
	24	<5	2010	Chiba	IPD	87.5		[51]
	17	<5	2011	Chiba	IPD	64.7		[51]
	16	<5	2012	Chiba	IPD	18.8		[51]
	17	<5	2013	Chiba	IPD	11.8		[51]
Singapore	180	Pediatric	1997–1999		IPD, non-IPD			[52]
	93	<19	1997–2004		IPD	78.1	92.3	[53]
	86	<13	2001–2006		IPD	85	96.5	[54]
South Korea	66	<5	1995–1998	Seoul	IPD, non-IPD	80.3		[55]
	140	<5	1999–2002	Seoul	IPD, non-IPD	56.4		[55]
	72	<5	2003–2005	Seoul	IPD, non-IPD	62.5		[55]
	13	<5	1996–1999	NS	IPD	69.2		[56]
	23	<5	2000–2003	NS	IPD	65.2		[56]
	36	<17	2002–2005	Seoul	RI	38.9	77.8	[57]
	72	<5	2003–2005	Seoul	IPD, non-IPD	62.5		[55]
	38	<5	2004–2006	NS	IPD	53.6		[56]
		<5	2006–2008	NS	IPD	50.0		[56]
	205	<18	2009–2010	Seoul	RI	16.6		[58]
	167	<5	2009–2010	Seoul	RI	30.5	53.9	[59]
	140	<18	2006–2010	4 cities (Seoul, Incheon, Ansan, Jeonu)	IPD	45	77.2	[60]
Taiwan	169	2–13	1997–2003	Kaoshiung	RI	86.4		[61]
	144	2–13	1997–2003	Kaoshiung	IPD, non-IPD	92.4		[61]
	286	≤14	1999–2004	Nationwide	IPD	85.0		[62]
	105	<15	1998–2010	Tainan	IPD	71.4		[63]
	68	<15	2004–2006	4 cities (Taipei, Taichung, Hualien, Kaohsiung)	IPD	83.8		[64]
	34	<2	2007	Nationwide	IPD	64.7	82.4	[65]
	89	2–4	2007	Nationwide	IPD	73	93.1	[65]
	26	5–9	2007	Nationwide	IPD	73.1	88.5	[65]
	35	Pediatric	2009–2011	Taipei	AOM			[66]
	42	<12	2009–2012	Taipei	IPD	37.8	79.6	[67]
Thailand	49	<19	1997–2000	Bangkok	NS			[68]
	256	<5	2003–2004	Sa Kaeo	Influenza-like	54.7		[69]
	58	<5	2003–2004	Sa Kaeo	Pneumonia	62.0		[69]
	115	<5	2000–2005	Nationwide	IPD	73.9	87.8	[70]
	19	<5	2005–2007	Sa Kaeo, Nakhon Phanom	Pneumonia	78.9	94.7	[71]
	14	<18	2004–2008	Bangkok	IPD			[72]
	64	<5	2006–2009	3 Provinces (Bankok, Nakorn Pratom, Nonthaburi)	IPD	70.3	81.2	[73]
	42	<5	2006–2009	3 Provinces (Bankok, Nakorn Pratom, Nonthaburi)	Non-IPD	61.9	76.2	[73]
	39	<5	2005–2010	Sa Kaeo, Nakhon Phanom	Pneumonia		92.3	[74]

^1^ NS: not specified; ^2^ RI: non-specified respiratory illness; ^3^ IPD: invasive pneumococcal disease; ^4^ AOM: acute otitis media.

**Table 2:** Predominant serotypes (>5%) in pre-and post PCV7 (7-valent pneumococcal conjugate vaccine) periods in East and Southeast Asian Countries.

Country	Pre-PCV7	Post-PCV7
**China**	19F (27.6%), 23F (11.7%), 6B (8.3%), 19A (8.0%), 14 (7.3%)	19F (23.2%), 19A (15.9%), 23F (12.9%), 14 (12.0%), 6B (10.9%)
**Hong Kong**	14 (34.2%), 6B (19.8%), 23F (17.6%), 19F (10.1%), 18C (5.0%)	6B (16.8%), 19F (16.6%), 23F (10.6%),14 (10.2%)
**Japan**	6B (21.9%), 19F (16.1%), 23F (14.4%), 14 (10.0%), 6A (6.7%)	6B (21.0%), 19A (13.0%), 23F (10.9%), 19F (8.7%), 14 (7.1%)
**Singapore**	19F (31.1%), 23F (22.3%), 14 (17.9%), 6B (12.85%)	
**South Korea**	23F (21.5%), 19F (20.7%), 19A (12.8%), 6A (8.3%), 6B (8.3%), 14 (7.0%), 9V (5.4%)	19A (18.5%), 19F (12.1%), 6B (10.1%), 6A, (7.1%), 23F (5.5%)
**Taiwan**	23F (23.7%), 14 (23.2%), 19F (19.1%), 6B (16.7%)	19A (24.9%), 14 (16.6%), 19F (15.7%), 6B (13.1%), 3 (6.5%), 23F (6.6%)
**Thailand**	6B (19.0%), 23F (17.3%), 19F (11.5%), 14 (9.8%)	23F (20%), 6B (18,6%), 14 (15.8%), 19F (11.7%), 19A (6.9%)

**Table 3:** Pneumococcal conjugate vaccine serotype coverage in East and Southeast Asian countries.

Country	Isolates (*n*)	Serotype Coverage (%)		
		PCV7	PCV10	PCV13
**China**	2,266 ^1^	57.6	59.9	70.7
	466 ^2^	60.5	60.9	79.8
**Hong Kong**	278 ^1^	89.2	89.2	91.3
	1,515 ^2^	55.6	55.6	66.1
**Japan**	1,603 ^1^	68.6	69.6	83.5
	676 ^2^	53.1	53.4	71.9
**Singapore**	273 ^1^	85.7	86.1	93.0
**South Korea**	242 ^1^	64.5	64.9	86.0
	622 ^2^	34.6	35.5	61.7
**Taiwan**	599 ^1^	87.1	87.3	92.0
	229 ^2^	54.1	58.1	89.5
**Thailand**	478 ^1^	74.1	76.6	81.8
	145 ^2^	68.3	69.0	82.8

^1^ The pre-PCV7 period; ^2^ the post-PCV7 period.
